# Staged Treatment of Cutaneous Calcinosis with Polylactic Acid Matrix, Negative Pressure Wound Therapy and Skin Grafting: A Case Report

**DOI:** 10.3390/reports9030301

**Published:** 2026-09-06

**Authors:** Paulina González-Aguilar, Mario Aurelio Martínez-Jiménez, Arturo Ortiz-Álvarez, Jose L. Ramirez-Garcialuna

**Affiliations:** 1 Wound Clinic, Regional High-Specialty Hospital “Ignacio Morones Prieto”, San Luis Potosí 78220, Mexico; pgonzalezagui@gmail.com; 2 Infectology Department, Regional High-Specialty Hospital “Ignacio Morones Prieto”, San Luis Potosí 78220, Mexico; cirugia.uaslp@gmail.com; 3 Division of Experimental Surgery, Faculty of Medicine, McGill University, Montreal, QC H3G 1A4, Canada; jose.ramirezgarcialuna@mail.mcgill.ca

**Keywords:** calcinosis cutis, arthritis, rheumatoid, negative-pressure wound therapy, poly(lactide), wound healing, debridement, case report

## Abstract

Background and Clinical Significance: Dystrophic calcinosis cutis involves calcium deposition in damaged or chronically inflamed tissues despite normal calcium and phosphate metabolism. Although typically associated with systemic sclerosis and dermatomyositis, it is rare in rheumatoid arthritis. Ulceration over calcified deposits may impair healing and function. This case highlights a staged reconstructive strategy to remove calcified tissue and achieve definitive wound closure. Case Presentation: A 73-year-old woman with a 22-year history of rheumatoid arthritis presented with painful, partially confluent pretibial ulcerations, purulent drainage, impaired mobility, and extrusion of chalk-like material. Radiographic and histopathological findings supported dystrophic calcinosis cutis. Deep-tissue culture grew *Escherichia coli*, treated with intravenous ertapenem. Tangential excision of necrotic tissue and accessible calcific deposits resulted in an irregular full-thickness defect extending to the pretibial fascia. A synthetic polylactic acid matrix was applied with single-use negative pressure wound therapy for 14 days, followed by split-thickness skin grafting on day 21. Graft take exceeded 90%, and residual areas epithelialized without regrafting. At 12 months, the wound remained completely epithelialized without recurrent ulceration, with pain resolution and return to baseline activities. Conclusions: In this patient with ulcerated dystrophic calcinosis cutis associated with rheumatoid arthritis, staged reconstruction using surgical excision, a polylactic acid matrix, single-use negative pressure wound therapy, and split-thickness skin grafting facilitated recipient-bed preparation and definitive cutaneous closure of the post-excisional defect. This case supports the feasibility of this sequence for wound reconstruction in selected complex ulcerated lesions. Further studies are needed to define its comparative role.

## 1. Introduction and Clinical Significance

Rheumatoid arthritis (RA) is a common chronic inflammatory disease, affecting approximately 0.5–1.0% of the adult population, whereas dystrophic calcinosis cutis in this setting remains exceptionally rare [1]. Calcinosis cutis is commonly associated with connective tissue diseases such as systemic sclerosis and dermatomyositis, while its occurrence in RA has been described less frequently [2,3]. In these cases, localized mineral deposition occurs within chronically inflamed tissues despite normal systemic calcium and phosphate levels. Clinically, calcinosis cutis may progress from indurated subcutaneous deposits to painful ulceration [4,5].

Once ulceration occurs, treatment becomes particularly challenging. Pharmacologic therapies for calcinosis cutis have variable and often limited and delayed benefits, while persistent calcium deposits act as a mechanical and inflammatory barrier. For localized lesions complicated by pain, infection, or disability, surgical removal of the calcified burden is often required; however, standard excision typically leaves a compromised, poorly vascularized defect that is not immediately suitable for definitive reconstruction [2,3,6].

Clinical Significance: This case report describes an uncommon presentation of ulcerated dystrophic calcinosis cutis associated with RA and highlights an integrated reconstructive strategy aimed not only at removing calcified deposits, but also at restoring conditions that allow progression toward definitive wound closure. The staged approach included surgical excision, polylactic acid (PLA) dermal matrix application with adjunctive single-use negative pressure wound therapy (sNPWT), followed by definitive coverage with split-thickness skin grafting.

## 2. Case Presentation

A 73-year-old woman with a 22-year history of RA and arterial hypertension presented to our wound care clinic with a 3-year history of progressive lesions on the anterior left pretibial region. The condition began as indurated subcutaneous nodules associated with intermittent erythema, edema, and tenderness. During the second year, the area progressively deteriorated, developing recurrent whitish drainage and extrusion of firm chalk-like material. Pain increased to 9/10 on the visual analog scale and was accompanied by worsening mobility beyond her baseline rheumatoid arthritis limitation. She denied prior local trauma, surgery, previous ulceration, or similar lesions elsewhere.

Her current medications included prednisone, leflunomide, and etoricoxib for rheumatoid arthritis, cholecalciferol supplementation, telmisartan for arterial hypertension and diltiazem 60 mg daily. Diltiazem had been initiated by her rheumatologist approximately 3 years before presentation as adjunctive systemic therapy directed at the underlying calcific process.

On examination, the anterior left pretibial region showed multiple irregular, partially confluent ulcerations distributed longitudinally over an area of indurated and hyperpigmented skin. The defects varied in size and were separated by bridges of chronically altered tissue, with the largest ulcerated area located distally. Multiple yellow-white calcific deposits and chalk-like material were visible within the wound beds, together with areas of devitalized tissue and exudate. Focal perilesional erythema, malodor, and purulent drainage were also present. Collectively, the ulcerated areas measured 8.0 cm × 6.0 cm and had a total surface area of 34.2 cm^2^ by calibrated digital planimetry using the Swift Skin and Wound™ application and Swift HealX™ calibration marker (Swift Medical Inc., Toronto, ON, Canada). No fascia, tendon, periosteum, or bone was exposed. No additional calcified nodules or ulcerations were identified elsewhere.

The dorsalis pedis and posterior tibial pulses were palpable bilaterally. The ankle–brachial index of the affected limb was 1.04, and arterial Doppler ultrasonography demonstrated triphasic flow without hemodynamically significant arterial obstruction. These findings indicated adequate perfusion for the planned reconstructive procedure, and no vascular intervention was considered necessary.

The patient had no history of diabetes mellitus or tobacco use. Her body mass index was 24.1 kg/m^2^. Hemoglobin was 12.3 g/dL, serum albumin was 3.9 g/dL, fasting glucose was 94 mg/dL, and glycated hemoglobin was 5.6%, without evidence of clinically significant anemia, hypoalbuminemia, or impaired glycemic control.

**Figure 1:**
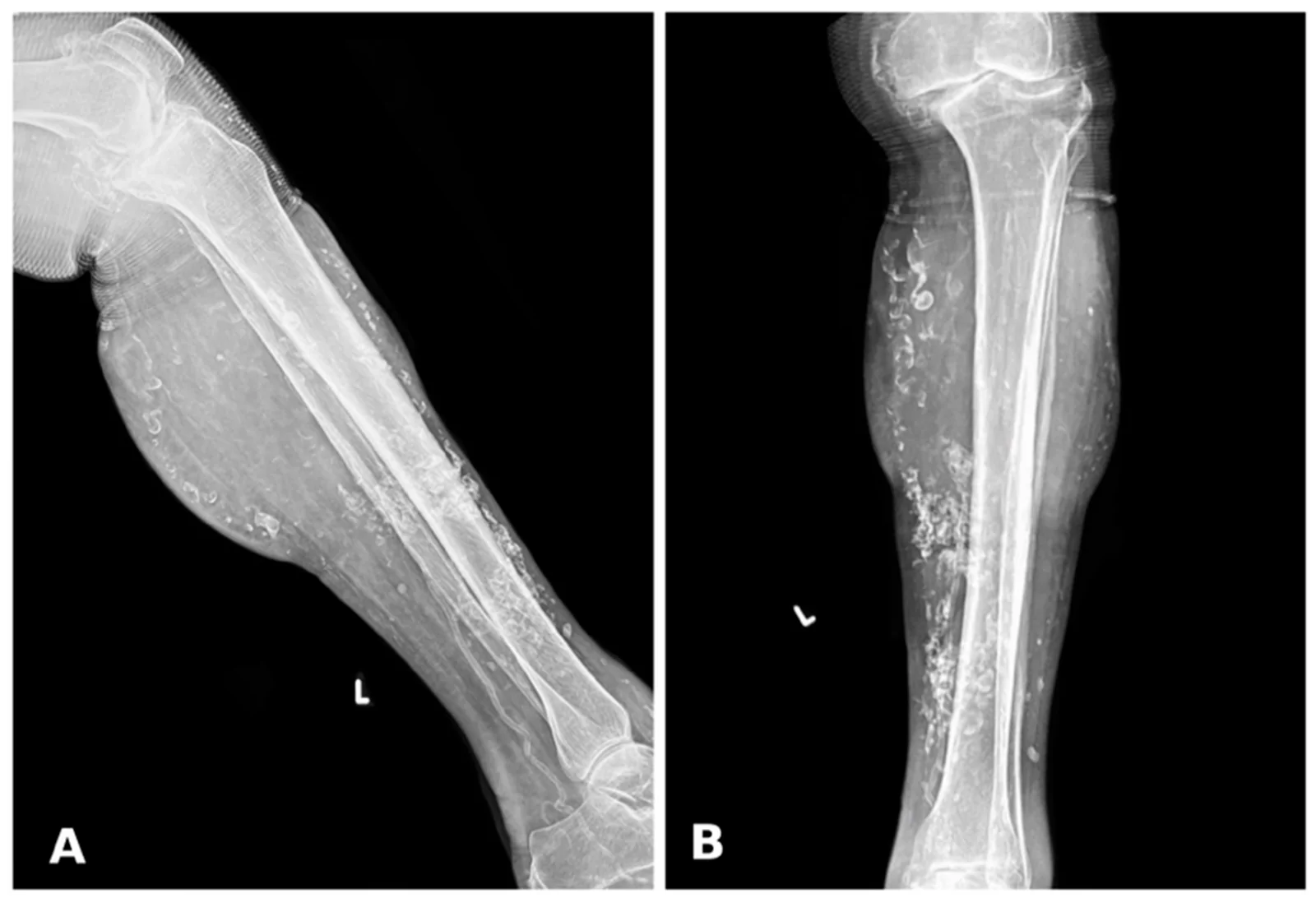
Radiographs of dystrophic calcinosis cutis of the lower left limb. Oblique (**A**) and anteroposterior (**B**) projections show multiple irregular calcifications distributed throughout the subcutaneous tissues. L: left.

Additional laboratory studies showed serum calcium of 9.2 mg/dL, phosphate of 3.4 mg/dL, alkaline phosphatase of 88 U/L, intact parathyroid hormone of 42 pg/mL, 25-hydroxyvitamin D of 32 ng/mL, serum creatinine of 0.78 mg/dL, and an estimated glomerular filtration rate of 78 mL/min/1.73 m^2^. Inflammatory markers were elevated, with a C-reactive protein level of 18 mg/L and an erythrocyte sedimentation rate of 48 mm/h. Creatine kinase was within the reference range at 72 U/L. Rheumatologic testing showed a rheumatoid factor level of 185 IU/mL and anti-cyclic citrullinated peptide antibodies above 250 U/mL. Plain radiographs of the left lower limb demonstrated multiple dense, irregular soft-tissue calcifications adjacent to the ulcerated defect (Figure 1).

A deep-tissue specimen was obtained using a sterile technique and submitted for aerobic culture and antimicrobial susceptibility testing. Culture grew *Escherichia coli* susceptible to ertapenem. When interpreted together with the clinical findings described above, the microbiological result supported the diagnosis of wound infection. Culture-directed treatment with ertapenem 1 g intravenously every 24 h was administered for a total of 10 days, including 7 days before surgery and 3 days postoperatively. After the initial 7 days of treatment, the local inflammatory findings had improved, and the patient remained clinically stable without progressive cellulitis or systemic manifestations of infection.

After initial antimicrobial therapy, manual tangential excision was performed with a scalpel under general anesthesia. Tissue viability was assessed according to color, consistency, adherence to the underlying planes, and preservation of viable pretibial fascia. Excision included the ulcerated tissue, nonviable wound margins, devitalized subcutaneous tissue, and macroscopically identifiable calcific deposits embedded within the wound bed. Resection proceeded through the full thickness of the skin and subcutaneous tissue to the level of the pretibial fascia, which appeared viable and was preserved. No bone, tendon, or periosteal exposure was identified. Debridement was continued until no residual purulence, necrotic tissue, or readily accessible calcific material remained. Hemostasis was achieved using bipolar electrocautery and sterile gauze compression. The resulting irregular full-thickness pretibial defect measured 11.5 cm × 4.5 cm at its maximum orthogonal dimensions, with a wound area of 40.4 cm^2^.

**Figure 2:**
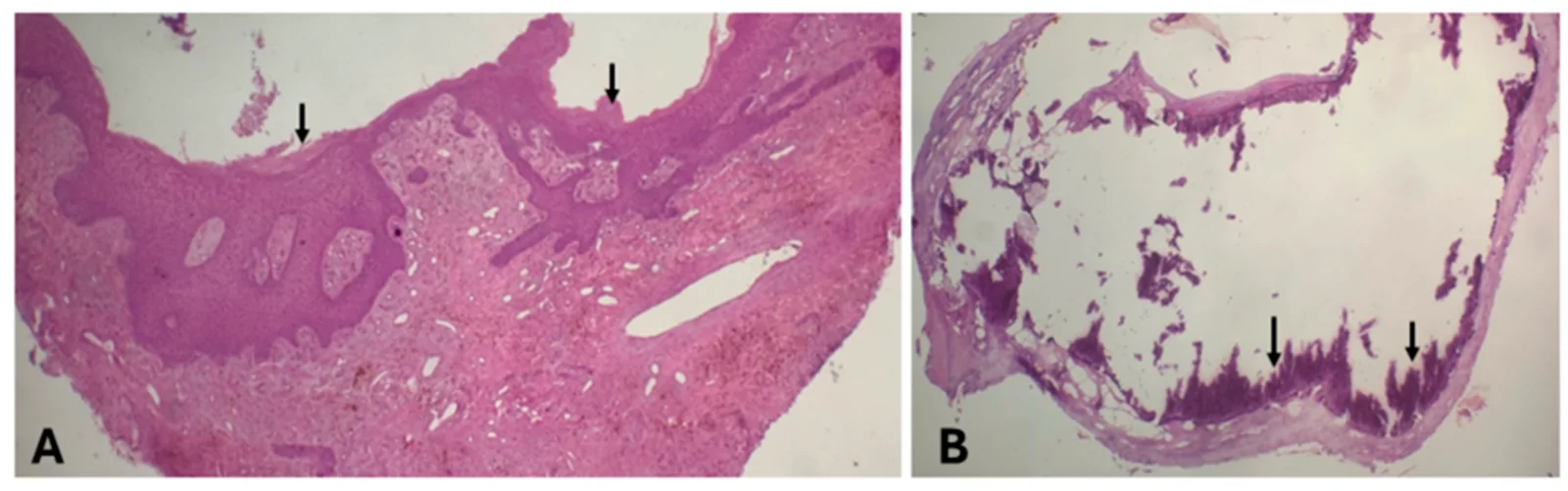
Histopathological findings of dystrophic calcinosis cutis. (**A**) Hematoxylin and eosin staining showing irregular epidermal acanthosis and surface disruption at the ulcer margin (arrows), with underlying dermal remodeling. (**B**) Extensive deposits of intensely basophilic amorphous material within fibrous soft tissue (arrows), consistent with calcium deposition.

The excised tissue was submitted for histopathological examination. Hematoxylin- and eosin-stained sections showed marked irregular epidermal acanthosis and a well-circumscribed basophilic calcific deposit surrounded by fibroconnective tissue. Vascular ectasia and hemosiderin deposition were present in the papillary and reticular dermis, consistent with chronic tissue injury and reparative change. No granulomatous foreign-body reaction, osteoid formation, vascular thrombosis, or malignant process was identified. These findings confirmed cutaneous calcium deposition and, together with the clinical and radiographic findings and normal mineral metabolism, supported the diagnosis of dystrophic calcinosis cutis (Figure 2).

Calciphylaxis was considered unlikely because renal function and mineral metabolism were normal and no thrombotic dermal vasculopathy was identified histologically. Tumoral calcinosis was not supported by the normal phosphate level or the absence of a periarticular lobulated mass. Gouty tophi, heterotopic ossification, and a foreign-body reaction were excluded by the absence of urate-type deposits, osteoid or mature bone formation, and polarizable foreign material, respectively.

**Figure 3:**
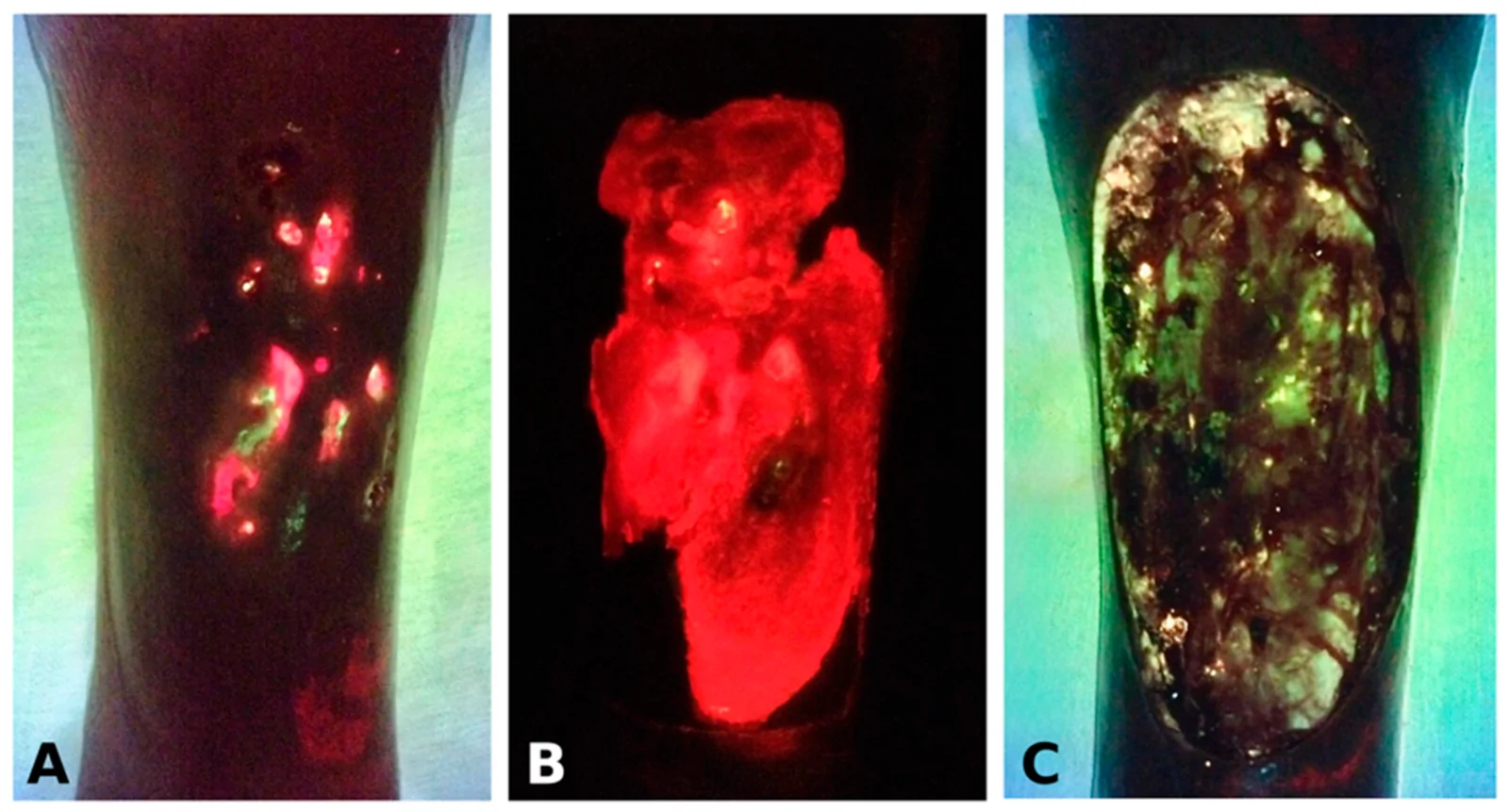
Intraoperative point-of-care fluorescence imaging before and after excision. Images were obtained using the MolecuLight i:X^®^ device (MolecuLight Inc., Toronto, ON, Canada). Under violet-light excitation, green represents endogenous tissue autofluorescence, whereas red fluorescence is associated primarily with bacterial porphyrins and does not independently confirm the presence of clinical infection [7]. (**A**) Initial assessment showing the spatial distribution of multifocal red fluorescence within the wound bed. (**B**) Persistent red fluorescence after superficial tissue removal. (**C**) Post-excisional assessment demonstrating a marked reduction in the distribution and intensity of the red signal. This reduction complemented the direct surgical assessment.

Point-of-care fluorescence imaging was performed before and after excision using the MolecuLight i:X^®^ device (MolecuLight Inc., Toronto, ON, Canada). Images were acquired under standardized dark conditions, following the manufacturer-recommended imaging procedure. Red fluorescence, associated primarily with bacterial porphyrins, was used to visualize their spatial distribution within the wound bed and complement the identification of areas requiring closer surgical assessment. The extent of excision was determined primarily by direct evaluation of tissue viability and the presence of necrotic tissue and calcific deposits. Post-excisional imaging demonstrated a marked reduction in red fluorescence, consistent with the intraoperative appearance of the prepared wound bed. Fluorescence findings were interpreted as complementary information and not as confirmation of microbiological eradication (Figure 3) [7].

Immediate split-thickness skin grafting was deferred because excision resulted in an irregular full-thickness pretibial defect with an initially nonuniform recipient bed. Recent infection, persistent local inflammation, and residual exudate were considered unfavorable for uniform graft-to-bed contact and early graft revascularization [8,9,10,11]. Definitive coverage was therefore postponed until the wound demonstrated uniform granulation and no clinical or microbiological evidence of persistent infection.

A sterile, single-use 18 cm × 18 cm SUPRA SDRM^®^ wound matrix (PolyMedics Innovations GmbH, Denkendorf, Germany) was used for staged wound-bed preparation. The fully synthetic, biodegradable, lactide-based matrix has a bimodal porous structure and a reported thickness of approximately 1.5–2.1 mm. The sheet was trimmed to conform to the dimensions and irregular contours of the post-excisional defect and placed directly over the wound surface. No sutures or staples were required for fixation.

A PICO 14 sNPWT system (Smith+Nephew, Hull, UK) dressing was applied over the matrix to maintain contact with the recipient bed and manage exudate. The system (Smith+Nephew, Hull, UK) operated continuously at a nominal negative pressure of −80 mmHg and was managed on an outpatient basis. The dressing remained in place without changes for the full 14-day treatment period, leaving the matrix–wound interface undisturbed. The dressing seal, exudate saturation, and device indicator were assessed during follow-up without removing the dressing. No clinically significant leakage, loss of negative pressure, excessive exudate accumulation, periwound maceration, bleeding, pain requiring device removal, or other device-related complications occurred. The system was removed as planned on day 14 [12].

After removal of the PICO system, no macroscopically visible remnants of the matrix were identified, consistent with advanced clinical resorption. The matrix had not required surgical removal. The underlying wound bed showed uniform granulation, allowing a deep-tissue specimen to be obtained for repeat culture before definitive coverage. The culture showed no bacterial growth.

The negative culture, uniform granulation, controlled exudate, and absence of purulent drainage, progressive erythema, cellulitis, or systemic manifestations of infection supported proceeding with STSG on day 21.

A 0.3-mm unmeshed STSG was harvested from the right anterolateral thigh using a Zimmer Air Dermatome. The graft was adapted to the recipient bed and secured to the wound margins with surgical staples. Petrolatum-impregnated gauze was placed directly over the graft and covered with sterile secondary gauze. The treated limb was elevated, and activity was restricted over 5 postoperative days to minimize shear across the graft–wound interface. The first graft inspection was performed on postoperative day 5, followed by dressing changes every 48 to 72 h until complete epithelialization. The staples were removed on postoperative day 14. No hematoma, seroma, graft-site infection, clinically significant partial graft loss, or other graft-related complication occurred.

The donor site was covered with petrolatum-impregnated gauze and an absorbent secondary dressing and achieved complete epithelialization within 14 days, without infection, delayed healing, or other donor-site complications.

At 7 weeks, graft take was estimated to be greater than 90%, with residual areas that subsequently epithelialized without regrafting. Wound-related pain had decreased from 9/10 to 3/10, with improved tolerance of standing and ambulation. By 2 months, the patient no longer reported pain and did not require additional rescue analgesia, although baseline etoricoxib therapy for rheumatoid arthritis was continued. The wound no longer imposed an additional limitation on ambulation, and the patient returned to her routine daily activities.

**Figure 4:**
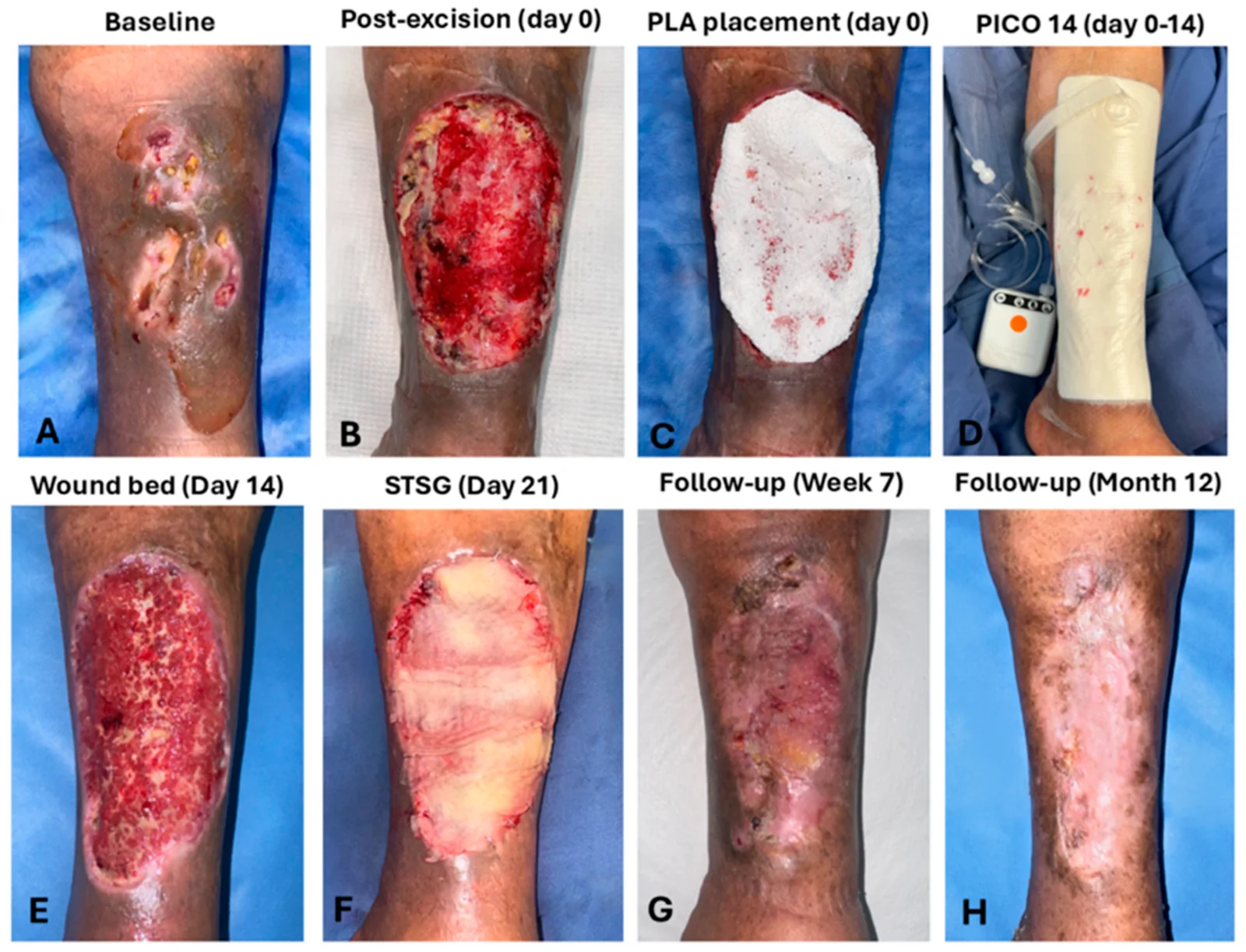
Clinical evolution of the ulcerated dystrophic calcinosis cutis and staged reconstruction. (**A**) Baseline appearance of the multiple irregular and partially confluent pretibial ulcerations, measuring collectively 8.0 cm × 6.0 cm, with a digitally measured surface area of 34.2 cm^2^. (**B**) Irregular full-thickness post-excisional defect on day 0, measuring 11.5 cm × 4.5 cm at its maximum orthogonal dimensions, with a surface area of 40.4 cm^2^. (**C**) Placement of the SUPRA SDRM^®^ polylactic acid matrix over the post-excisional defect on day 0. (**D**) Application of the PICO 14 single-use negative pressure wound therapy system, maintained from day 0 through day 14. (**E**) Uniformly granulating wound bed on day 14 after removal of the PICO system and advanced clinical resorption of the matrix. (**F**) Placement of a 0.3-mm unmeshed split-thickness skin graft secured with surgical staples on day 21. (**G**) Greater than 90% graft take at 7 weeks, with small residual areas undergoing epithelialization. (**H**) Completely epithelialized and stable reconstructed area at 12 months, without drainage, recurrent ulceration, or clinically evident new calcific deposits. All photographs show the anterior left pretibial region in a consistent orientation, with the proximal aspect at the top and the distal aspect at the bottom. Day 0 corresponds to the date of surgical excision and matrix placement.

At 12 months, the reconstructed area remained completely epithelialized and stable, without drainage or recurrent ulceration. Clinical inspection and palpation revealed no visible or palpable new calcific deposits. The scar was predominantly flat and pliable, with residual pigmentary and surface irregularity but without clinically significant hypertrophy, contracture, adherence to deeper tissues, drainage, or tenderness on palpation (Figure 4). Diltiazem remained unchanged throughout the reconstructive treatment and was still being administered at the 12-month follow-up.

The patient reported that wound closure relieved the pain associated with the ulcer and allowed her to return to her pre-ulcer level of mobility and daily activities. She expressed satisfaction with the functional and clinical outcome (Table 1).

## 3. Discussion

Ulcerated dystrophic calcinosis cutis presents two interrelated but therapeutically distinct challenges: persistent deposition of insoluble calcium salts within chronically damaged tissue and the development of a wound characterized by inflammation, transepidermal extrusion of chalk-like material, pain, impaired healing, and susceptibility to secondary infection. This distinction is clinically relevant because pharmacologic therapies are directed primarily at the calcific process, whereas established ulceration, tissue necrosis, and infection frequently require local wound management or surgical intervention [2,5].

Several systemic and local pharmacologic agents—including diltiazem, bisphosphonates, minocycline, colchicine, and sodium thiosulfate—have been used for calcinosis cutis [2,5]. Diltiazem is among the most frequently reported systemic treatments, although its use remains off-label and the available evidence is heterogeneous. Palmieri et al. reported clinical and radiographic reduction in calcific lesions in four patients with idiopathic or CREST-associated calcinosis treated with 240–480 mg/day for 1–12 years [13]. Subsequent retrospective studies showed less consistent responses: partial improvement occurred in 9 of 17 patients with autoimmune connective-tissue diseases [5] and in 5 of 8 patients receiving lower-dose therapy [14]. Conversely, Vayssairat et al. observed only slight radiographic regression in 3 of 12 patients with systemic sclerosis despite prolonged follow-up [15]. Collectively, these findings suggest that diltiazem may benefit selected patients, but the magnitude and durability of response remain unpredictable, and its ability to control advanced ulcerated disease has not been established.

Surgical excision provides the most direct method of reducing the local calcific burden and is generally considered for lesions associated with pain, ulceration, recurrent infection, or functional impairment. In a retrospective cohort of calcinosis associated with autoimmune connective-tissue diseases, excision produced a complete response in 22 of 28 patients and a partial response in five [5]. Similarly, Róbert et al. reported at least a partial response in all seven surgically treated patients with available follow-up [14]. However, surgical outcomes appear to depend on lesion distribution and the feasibility of complete removal. Lapner and Goetz evaluated debulking in nine patients with scleroderma-associated digital calcinosis and documented recurrence in seven, including three early complete recurrences. Six patients experienced complications, including weakness, reduced motion, numbness, and superficial infection [16]. Patients with one or two discrete deposits had better outcomes than those with diffuse involvement. A systematic review of surgically treated soft-tissue calcifications similarly reported a 13.3% recurrence rate after simple excision, whereas no recurrence was documented after radical excision. More extensive resections, however, frequently required dermal matrices or flap coverage [17]. Excision alone is therefore most applicable to discrete lesions that can be completely removed and closed primarily. In diffuse or ulcerated disease, the benefit of more extensive calcium removal must be balanced against wound-healing complications, functional morbidity, and the anticipated reconstructive requirements.

Following excision, conventional wound-bed preparation with serial debridement, infection control, exudate management, and moist dressings remains the most accessible and least technologically dependent approach. It may be sufficient when the defect is superficial, adequately vascularized, and capable of developing uniform granulation without structural support. However, prolonged open-wound care may require frequent dressing changes, and persistent calcific deposits, inflammation, or nonuniform vascularity may delay progression toward definitive closure.

Dermal substitutes provide an intermediate option when primary closure or immediate grafting is not appropriate. Available products include collagen-based, acellular dermal, placental, fish-skin, and fully synthetic matrices, and their selection depends on wound depth, exposed structures, infection status, handling requirements, accessibility, and cost. In calcinosis cutis, Johnson et al. reported complete healing maintained for 18 months after wide excision, acellular dermal matrix placement, and NPWT [18]. PLA matrices offer biodegradability, conformability, and avoidance of donor-derived components; they support cellular activity, collagen deposition, angiogenesis, and moisture regulation [19]. Comparative studies in diabetic foot and venous leg ulcers have reported favorable healing outcomes with PLA relative to collagen dressings and intact fish-skin grafts [20,21]. Nevertheless, these studies involved different wound etiologies, and no evidence establishes the superiority of PLA or any other dermal substitute following calcinosis excision. PLA was selected because its conformability and biodegradability were considered suitable for temporary coverage of the irregular post-excisional defect and staged development of a uniformly granulating recipient bed without requiring subsequent surgical removal of the matrix [22].

NPWT and flap reconstruction occupy different roles within the reconstructive pathway. NPWT is primarily a temporizing modality used to manage exudate and promote granulation before closure; successful staged use followed by skin grafting has been reported in pretibial calcinosis [23,24]. NPWT without a matrix may be sufficient for an adequately perfused defect capable of developing uniform granulation without additional structural support and avoids the added cost of a dermal substitute. However, no comparative evidence currently establishes whether combining PLA with NPWT provides an advantage over NPWT alone following calcinosis excision. Skin-graft survival depends on a clean, stable, and adequately vascularized recipient bed that permits uniform graft contact [8]. This is particularly relevant in the lower extremity, where Reddy et al. reported failure in approximately one-third of 70 skin grafts and identified peripheral vascular disease and immunosuppressive therapy as significant risk factors [11]. Bacterial colonization has also been identified as a significant predictor of graft loss [10].

In the present case, each intervention served a distinct therapeutic objective. Excision reduced the local burden of necrotic tissue and accessible calcific deposits. PLA matrix placement and sNPWT then provided a bridge to definitive reconstruction by stabilizing the matrix–wound interface, controlling exudate, and allowing formation of a uniform granulating recipient bed. Split-thickness skin grafting subsequently achieved definitive cutaneous coverage. This sequence addressed the local consequences of ulcerated calcinosis and the resulting tissue defect, rather than the biological predisposition to calcium deposition. Diltiazem was continued for the underlying calcific process.

This report has several limitations. Although PLA matrix is indicated for the management of full-thickness and surgical wounds, evidence specifically evaluating its use following excision of calcinosis cutis remains limited [18,19,22]. The interpretation of this outcome is limited by the single-patient design, absence of a comparator and concurrent use of multiple interventions. Therefore, neither wound closure nor the absence of clinically evident recurrence can be attributed to any individual treatment component. These findings support the feasibility of this reconstructive sequence in a complex ulcerated lesion but do not establish its comparative effectiveness. Further evaluation in additional patients is required.

## 4. Conclusions

The management of ulcerated dystrophic calcinosis cutis requires an individualized and multimodal approach. In this case, staged reconstruction with surgical excision, PLA matrix placement, and sNPWT, followed by STSG, allowed preparation of the recipient bed and definitive coverage of the post-excisional defect. Complete re-epithelialization was accompanied by reduced pain and a progressive return to baseline daily activities.

## Figures and Tables

**Table 1:** Clinical timeline of the staged management and follow-up. Day 0 corresponds to surgical excision, PLA matrix placement, and initiation of sNPWT. IV, intravenous; PLA, polylactic acid; sNPWT, single-use negative pressure wound therapy.

Time Point	Clinical Event
3 years before presentation	Pretibial nodules developed. Diltiazem 60 mg/day was initiated as adjunctive therapy for the calcific process by rheumatologist.
Second year of lesion evolution	The lesions progressed to painful ulcerations with recurrent whitish drainage and extrusion of chalk-like material.
Initial assessment	Multiple partially confluent ulcers measured collectively 8.0 × 6.0 cm (34.2 cm^2^). Pain was 9/10 with worsening mobility.
Preoperative period	A sterile deep-tissue culture grew *Escherichia coli* susceptible to ertapenem. Ertapenem 1 g IV every 24 h was administered for 7 days before surgery.
Day 0	Tangential excision of nonviable tissue and accessible calcific deposits resulted in a full-thickness defect measuring 11.5 × 4.5 cm (40.4 cm^2^). A PLA matrix and PICO 14 sNPWT were applied.
Days 0–3	Postoperative ertapenem was continued for 3 days, completing a total 10-day course.
Day 14	PICO was removed. The wound showed uniform granulation and clinical matrix resorption. Repeat deep-tissue culture showed no bacterial growth.
Day 21	A 0.3-mm unmeshed split-thickness skin graft was applied.
Week 7	Graft take was >90%; residual areas epithelialized without regrafting. Pain decreased to 3/10 and mobility improved.
Month 12	Stable closure was maintained without drainage, recurrent ulceration, or clinically evident new calcific deposits. The scar remained predominantly flat and pliable.

## Data Availability

The original contributions presented in this study are included in the article. Further inquiries can be directed to the corresponding author.

## Abbreviations

The following abbreviations are used in this manuscript:

RA	Rheumatoid arthritis
PLA	Polylactic Acid
sNPWT	Single-use Negative Pressure Wound Therapy
